# Implementation of a frailty screening programme and Geriatric Assessment Service in a nephrology centre: a quality improvement project

**DOI:** 10.1007/s40620-020-00878-y

**Published:** 2020-10-10

**Authors:** Andrew C. Nixon, Julie Brown, Ailsa Brotherton, Mark Harrison, Judith Todd, Dawn Brannigan, Quinta Ashcroft, Beng So, Neil Pendleton, Leonard Ebah, Sandip Mitra, Ajay P. Dhaygude, Mark E. Brady

**Affiliations:** 1 Department of Renal Medicine, Lancashire Teaching Hospitals NHS Foundation Trust, Royal Preston Hospital, Sharoe Green Lane, Preston, PR2 9HT UK; 2 Division of Cardiovascular Sciences, University of Manchester, Manchester, UK; 3 Centre for Health Research and Innovation, National Institute of Health Research Lancashire Clinical Research Facility, Lancashire Teaching Hospitals NHS Foundation Trust, Preston, UK; 4 Lancashire Teaching Hospitals Integrated Frailty Team, Lancashire Teaching Hospitals NHS Foundation Trust, Preston, UK; 5 Continuous Improvement Team, Lancashire Teaching Hospitals NHS Foundation Trust, Preston, UK; 6 Department of Business Intelligence, Lancashire Teaching Hospitals NHS Foundation Trust, Preston, UK; 7 Division of Neuroscience and Experimental Psychology, University of Manchester, Manchester, UK; 8 Department of Renal Medicine, Manchester University NHS Foundation Trust, Manchester, UK; 9 Manchester Academic Health Sciences Centre, University of Manchester, Manchester, UK; 10 Devices for Dignity, National Institute of Health Research MedTech and In-vitro Diagnostics Co-operative, Sheffield, UK

**Keywords:** Aging, Chronic kidney disease, Elderly, End stage renal disease, Hemodialysis

## Abstract

**Introduction:**

The aims of this quality improvement project were to: (1) proactively identify people living with frailty and CKD; (2) introduce a practical assessment, using the principles of the comprehensive geriatric assessment (CGA), for people living with frailty and chronic kidney disease (CKD) able to identify problems; and (3) introduce person-centred management plans for people living with frailty and CKD.

**Methods:**

A frailty screening programme, using the Clinical Frailty Scale (CFS), was introduced in September 2018. A Geriatric Assessment (GA) was offered to patients with CFS ≥ 5 and non-dialysis- or dialysis-dependent CKD. Renal Frailty Multidisciplinary Team (MDT) meetings were established to discuss needs identified and implement a person-centred management plan.

**Results:**

A total of 450 outpatients were screened using the CFS. One hundred and fifty patients (33%) were screened as frail. Each point increase in the CFS score was independently associated with a hospitalisation hazard ratio of 1.35 (95% CI 1.20–1.53) and a mortality hazard ratio of 2.15 (95% CI 1.63–2.85). Thirty-five patients received a GA and were discussed at a MDT meeting. Patients experienced a median of 5.0 (IQR 3.0) problems, with 34 (97%) patients experiencing at least three problems.

**Conclusions:**

This quality improvement project details an approach to the implementation of a frailty screening programme and GA service within a nephrology centre. Patients living with frailty and CKD at risk of adverse outcomes can be identified using the CFS. Furthermore, a GA can be used to identify problems and implement a person-centred management plan that aims to improve outcomes for this vulnerable group of patients.

**Electronic supplementary material:**

The online version of this article (10.1007/s40620-020-00878-y) contains supplementary material, which is available to authorized users.

## Introduction

One of the greatest challenges for healthcare in the twenty-first century is population ageing [1]. With population ageing comes an increasing prevalence of individuals living with multimorbidity and associated frailty, the state of vulnerability to disproportionate deterioration in health status when exposed to stressor events [2, 3]. This is relevant for all areas of healthcare, but it is especially so for specialised services, such as nephrology, that care for people living with chronic conditions that appear to hasten the decline from fitness to frailty [4]. The prevalence of frailty is far greater in those with chronic kidney disease (CKD) compared to the general older population, with one study reporting a prevalence as high as 73% in dialysis-dependent CKD [5, 6]. Importantly, frailty is associated with an increased risk of adverse outcomes in people with CKD, including worse health-related quality of life (HRQOL) [7] and increased hospitalisation and mortality risk [5]. Despite the high prevalence of frailty and associated risk of adverse health outcomes, proactive frailty identification is not routine practice within nephrology services. Without systematic screening, frailty is unlikely to be recognised until individuals experience an adverse event that may have been preventable with the implementation of an evidence-based intervention [8].

A 2016 clinical practice guideline recommended a ‘simple score be used on a regular basis to assess functional status in older patients with CKD’, though it stated that there was ‘no consensus on the most appropriate tool for assessing physical function in older patients with advanced CKD’ [9]. Recognising that identifying patients living with CKD and frailty is the first step towards improving outcomes for this vulnerable group, a study was performed at our centre, Lancashire Teaching Hospitals NHS Foundation Trust (LTHTR), that evaluated the diagnostic accuracy of frailty screening methods in patients with CKD [10]. This study demonstrated that the Clinical Frailty Scale (CFS), which is a 9-point scale with descriptions for levels of frailty [11], is an accurate frailty screening method in adults with advanced CKD [10]. The question is then what can be done to improve outcomes once an individual is screened as frail? The Comprehensive Geriatric Assessment (CGA) has been shown to improve outcomes for older adults when performed during an acute hospital admission; specifically, older adults are more likely to be living in their own home one year after hospitalisation [12]. It is defined as ‘a multidimensional, multidisciplinary process which identifies medical, social and functional needs, and the development of an integrated/co-ordinated care plan to meet those needs’ and is now the accepted standard of care of the older patient living with frailty [8]. Although there is limited evidence in nephrology on how best to improve outcomes for patients living with frailty and CKD, studies have demonstrated that the CGA (or a modified version) is feasible within nephrology services and can be used to identify geriatric impairments in CKD populations [13]. Once problems are identified, management strategies can be developed that aim to improve outcomes. Considering the existing evidence, our aims were to: (1) proactively identify people living with frailty and CKD; (2) introduce a practical assessment, using the principles of the CGA, for people living with frailty and CKD able to identify problems; and (3) introduce person-centred management plans for people living with frailty and CKD.

## Methods

### Context

The LTHTR Department of Renal Medicine participated in the Specialised Clinical Frailty Network (SCFN) delivered by National Health Service (NHS) Elect between September 2018 and September 2019 [14]. The SCFN is a quality improvement collaborative that supports healthcare teams to explore how best to integrate frailty assessment and management into care pathways. Details of the improvement collaborative are fully described at https://www.scfn.org.uk. LTHTR was one of five nephrology sites that participated in this wave of the improvement programme. All sites met on five occasions over 12 months to discuss approaches to care improvement and share quality improvement experiences. A multi-professional development group was established within the LTHTR Department of Renal Medicine in September 2018. The group met regularly during all phases of the project and adopted the model for improvement framework [15]. The project discussed hereafter is based upon the work conducted at LTHTR. Supplementary Fig. 1 illustrates the project driver diagram. Supplementary Table 1 describes the Plan-Do-Study-Act (PDSA) cycles performed.

### Awareness, education and training

Education on the relevance of frailty for nephrology services and on how to use the CFS was provided via departmental presentations (September and November 2018) and ad-hoc one-on-one sessions. An animated video was created to provide education on frailty screening, to describe the purpose of the Renal Frailty MDT and how to refer to the planned service. It was circulated within the department and displayed on LTHTR screens. The multi-professional development group collaborated with the trust End of Life Care Educator who provided bespoke training to dialysis nursing staff on how to support advance care planning discussions with patients.

### Frailty screening programme

A frailty screening programme, using the CFS, was introduced within the department in September 2018. Screening was performed in LTHTR outpatient clinics, during clinical nurse specialist (CNS) home visits and on two LTHTR haemodialysis units by clinicians, CNS and dialysis staff nurses. The CFS was incorporated within a pre-existing LTHTR care tool, called the Holistic Care Tool, already used on the haemodialysis units quarterly. The Holistic Care Tool is used to assess patient functional status and psychological distress with a view to addressing problems that may otherwise remain unknown. CFS score documentation was incorporated within the trust electronic patient record system.

### Geriatric Assessment

A holistic home assessment was developed using the principles of the CGA. Though it was a multi-domain assessment, it was only performed by one healthcare professional (an occupational therapist [OT] with experience performing a CGA) and therefore was termed a Geriatric Assessment (GA). The domains that were assessed during the GA included depression/anxiety, falls risk, cognition, polypharmacy, continence, skin integrity, nutrition, activities of daily living (ADLs) and social issues.

*Depression and anxiety* The Public Health Questionnaire-9 (PHQ-9) and Generalised Anxiety Disorder-7 (GAD-7) measures were used to screen for depression and anxiety, respectively [16, 17]. If a patient scored ≥ 3 on the first two questions of the PHQ-9 or GAD-7, the respective measure was completed in full. Scores ≥ 10 were used to signify moderate symptoms of depression and anxiety and denote a problem in this domain [16, 17].

*Falls* Falls risk was assessed using the Falls Risk Assessment Tool (FRAT) [18]. Individuals that scored ≥ 3 on this tool were considered at risk of falls, which represented a problem in this domain. Patients were referred to a dedicated falls clinic if the OT felt further investigation into the cause of falls was needed.

*Cognition:* Initially, a simple question was used to screen for cognitive impairment: ‘Has the patient been more forgetful in the last 12 months to the extent that it has significantly affected their life?’. If the answer was ‘yes’ to this screening question, the 6-Item Cognitive Impairment Test (6-CIT) was performed [19]. Problems with executive function are a prominent feature of cognitive impairment in those with CKD [20]. The development group therefore later decided to incorporate the Montreal Cognitive Assessment (MoCA), which is a useful test of executive function [21], routinely within the GA. A score ≤ 24 was used to identify a problem in the cognition domain [22, 23]. With patient permission, a patient’s general practitioner was informed if there were concerns about cognitive impairment.

*Polypharmacy* Medications were reviewed and polypharmacy was defined as ≥ 5 prescribed medications [24].

*Continence and skin integrity* Patients who reported issues with continence or skin integrity were defined as having a problem with this domain.

*Nutrition* Patients who reported issues with nutrition or unintentional weight loss were defined as having a problem with this domain.

*Activities of daily living* ADLs were assessed by an OT during the consultation. ADLs were sub-categorised as basic ADLs (BADLs), i.e. self-care tasks, and intermediate ADLs (IADLs), i.e. tasks related to maintaining an independent household. Any reported issues performing ADLs represented a problem in this domain.

*Social issues* Social issues were defined as living circumstances that were considered to pose a risk to a patient’s well-being, e.g. main carer unwell and unable to meet patient’s care needs.

### Renal frailty multi-disciplinary team

A Renal Frailty MDT was established that included a clinician, dialysis sister, Kidney Choices CNS, dietitian, psychologist, OT and social worker. Any staff member in the Department could refer patients for a GA. A dedicated Renal Frailty MDT email account was established and used as a single point of access for referrers. Referral criteria were modified during the project using the PDSA approach. The final established referral criteria were: age ≥ 18 years, CFS ≥ 5 (or CFS < 5 with concerns about mobility, cognition or nutritional status) and non-dialysis or dialysis dependent CKD. Patients who received a GA were discussed at a MDT meeting during which problems and associated clinical needs were discussed. A person-centred management plan was created and the MDT considered the appropriateness of starting advance care planning discussions with patients. The outcomes of MDT meetings were summarised on tailor-made pro forma and shared with the wider nephrology team involved in the patient’s care.

### Measures

Screening data collected included number of patients screened each week (and individual CFS scores) between September 2018 and July 2019. Hospitalisation and mortality data were collected between September 2018 and September 2019 to assess the CFS’s ability to identify patients at risk of adverse outcomes. GA and MDT meeting data were collected between November 2018 and January 2020. Data collected included number and type of problems experienced by each patient. The rationale for doing so was to assess the ability of the GA to identify problems and thereby assess its utility. Data were collected on the number and type of actions recommended following GA and MDT meetings to assess the impact the new pathway may have on patient care.

### Statistical analysis

Quantitative data are presented as mean and standard deviation (SD) or median and interquartile range (IQR) depending on the distribution of the data. Normal *Q*–*Q* plots were visually inspected to assess data distribution. Categorical data are presented as frequencies and percentages. Differences in parametric data were assessed using the independent *t* test. Differences in non-parametric data were assessed using the Mann–Whitney *U* test. The chi square test was used to assess differences in categorical data. If test assumptions were not met, between group differences for categorical data were assessed using the Fisher’s exact test. Cox regression analyses were used to assess the time to first hospitalisation and mortality hazard ratios for each unit increase in CFS score, whilst adjusting for age, sex and for non-dialysis and dialysis dependent CKD variables. The assumption of proportional hazards was assessed for each model by reviewing the significance of each time-by-variable interaction. A *p* value < 0.05 was considered statistically significant. All statistical analyses were performed using IBM SPSS version 25 (IBM, USA).

## Results

### Frailty Screening Programme

**Fig. 1:**
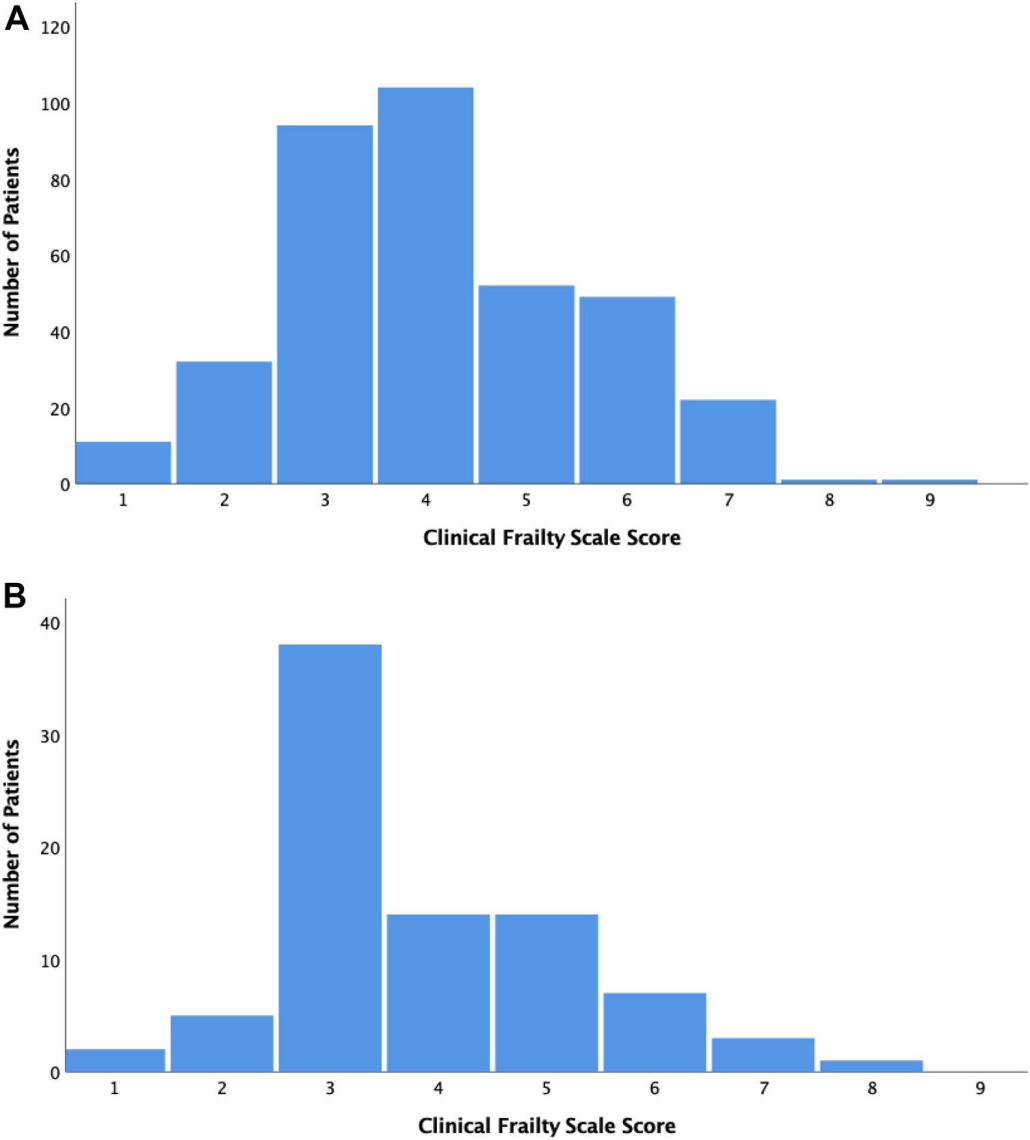
**a** Distribution of Clinical Frailty Scale scores for patients with non-dialysis dependent chronic kidney disease. **b** Distribution of Clinical Frailty Scale scores for patients with dialysis-dependent chronic kidney disease

A total of 450 outpatients (366 non-dialysis and 84 haemodialysis patients) were screened using the CFS. Only first screening episodes were included in the analysis. Figure 1 demonstrates the distribution of CFS Scores for patients with non-dialysis- and dialysis-dependent CKD. One hundred and fifty patients (33%) were screened as frail (i.e. CFS ≥ 5). Frail patients were older than non-frail patients (median age 81 years [IQR 14] vs. 74 years [IQR 11], *p* < 0.001). There was no significant difference in the proportion of female (46.7% [*n* = 70] vs. 44.0% [*n* = 132], *p* = 0.59) and dialysis-dependent patients (16.7% [*n* = 25] vs. 19.7% [*n* = 59], *p* = 0.44) categorised as frail and non-frail. The median follow-up time was 210 days (95% confidence interval [CI] 203–217). More frail patients were hospitalised (41.3% [*n* = 62] vs. 21.3% [*n* = 64], *p* < 0.001) and died (14.7% [*n* = 22] vs. 1.7% [*n* = 5], *p* < 0.001) than non-frail patients. Table 1 presents the results of the Cox regression analyses. When adjusted for age, sex and dialysis dependence, each point increase in CFS score was associated with a mortality hazard ratio of 2.15 (95% CI 1.63–2.85). There was a time interaction between the non-dialysis/dialysis variable and first hospitalisation, which was therefore included in the final ‘first hospitalisation’ model (denoted as time*dialysis). When adjusted for age, sex, dialysis dependence and time*dialysis, each point increase in CFS score was associated with a first hospitalisation hazard ratio of 1.35 (95% CI 1.20–1.53).

**Table 1:** Association between frailty and first hospitalisation/mortality for patients living with CKD

	Hazard ratio	95% Confidence interval
First hospitalisation		
Model 1		
CFS score (per point increase)	1.35	1.20–1.53
Age (per year increase)	1.00	0.98–1.01
Female	0.99	0.69–1.41
Dialysis dependent^a^	1.31	0.81–2.11
Model 2		
CFS score (per point increase)	1.35	1.20–1.53
Age (per year increase)	1.00	0.98–1.01
Female	0.96	0.68–1.37
Dialysis dependent	0.63	0.28–1.38
Time*dialysis^b^	1.01	1.00–1.01
Mortality		
CFS score (per point increase)	2.15	1.63–2.85
Age (per year increase)	1.01	0.96–1.05
Female	0.44	0.19–1.01
Dialysis dependent	0.75	0.23–2.46

*CFS* Clinical Frailty Scale

^a^Violates proportional hazards assumption

^b^Time interaction between the non-dialysis/dialysis variable and first hospitalisation

### Geriatric Assessment Service

Thirty-five patients received a GA and were discussed at a MDT meeting. Table 2 details non-dialysis- and dialysis-dependent patient demographics and clinical characteristics. The median age was 80 (IQR 19) years. There were 15 (43%) female patients who received a GA and 21 (60%) patients were receiving haemodialysis. Dialysis-dependent patients were younger than non-dialysis patients (median age 69.0 [IQR 17.5] vs. 83.5 [IQR 7.0] years, *p* < 0.001). The median CFS score was 6.0 (IQR 1.0). Thirty-two (91%) patients within this group had an OT-assessed CFS score ≥ 5.

**Table 2:** Demographics and clinical characteristics of patients that received a Geriatric Assessment

	Non-dialysis (*n* = 14)	Dialysis (*n* = 21)	All (*n* = 35)	*p* value
Age, years, median (IQR)	83.5 (7.0)	69.0 (17.5)	80.0 (19.0)	< 0.001
Female, *n* (%)	8 (57)	7 (33)	15 (43)	0.16
eGFR, ml/min/1.73 m^2^, mean (SD)	14.3 (5.7)	–	–	–
Underlying kidney disease				
Diabetes, *n* (%)	2 (14)	7 (33)	9 (26)	0.16
Renovascular/ischaemic, *n* (%)	7 (50)	4 (19)	11 (31)	
Other, *n* (%)	5 (36)	10 (48)	15 (43)	
Comorbidities				
Diabetes mellitus, *n* (%)	6 (43)	10 (48)	16 (46)	0.78
IHD, *n* (%)	3 (21)	8 (38)	11 (31)	0.46
Hypertension, *n* (%)	12 (86)	17 (81)	29 (83)	1.00
Stroke, *n* (%)	2 (14)	1 (5)	3 (9)	0.55
CCF, *n* (%)	1 (7)	2 (10)	3 (9)	1.00
COPD, *n* (%)	1 (7)	0 (0)	1 (3)	0.40
Dementia, *n* (%)	2 (14)	1 (5)	3 (9)	0.55
Active malignancy, *n* (%)	0 (0)	1 (5)	1 (3)	1.00
Medications, mean (SD)	8.0 (3.3)	10.0 (3.5)	9.0 (4.0)	0.27
Living arrangements				
With family/partner, *n* (%)	9 (64)	14 (67)	23 (66)	1.00
Alone, *n* (%)	5 (36)	6 (29)	11 (31)	
Care home resident, *n* (%)	0 (0)	1 (5)	1 (3)	
CFS Score, median (IQR)	6.0 (0.5)	6.0 (1.0)	6.0 (1.0)	0.19
PHQ-9 ≥ 10, *n* (%)*	1 (9)	7 (35)	8 (26)	0.20
GAD-7 ≥ 10, *n* (%)*	0 (0)	4 (20)	4 (13)	0.27
MoCA ≤ 24, *n* (%)**	2 (50)	8 (100)	10 (83)	0.09

Data presented as median (IQR), mean (± SD) or number (%)

*eGFR* estimated Glomerular Filtration Rate, *IHD* ischaemic heart disease, *CCF* congestive cardiac failure, *COPD* chronic obstructive pulmonary disease, *CFS* Clinical Frailty Scale, *MoCA* Montreal Cognitive Assessment

*Data available for 11 non-dialysis and 20 dialysis patients

**Data available for 4 non-dialysis and 8 dialysis patients

Patients experienced a median of 5.0 (IQR 3.0) problems, with 34 (97%) patients experiencing at least three problems. There were more problems experienced by dialysis-dependent patients compared to non-dialysis patients (6.0 [IQR 1.5] vs 3.5 [IQR 2.0], *p* = 0.007). The median number of recommended actions was 4.0 (IQR 2.0), with more actions recommended for dialysis-dependent patients compared to non-dialysis patients (5.0 [IQR 2.0] vs. 3.0 [IQR 2.0], *p* = 0.004). Table 3 details the specific problems experienced by non-dialysis- and dialysis-dependent patients and the associated recommended actions. All patients had problems with IADLs. Twenty-six (74%) patients had at least one fall in the 12 months preceding GA and 32 (91%) patients were considered at risk of future falls. Thirteen (37%) patients had cognitive impairment. However, 10 out of the 12 patients that had a MoCA performed had a score ≤ 24. All patients received health and social care advice, for example falls prevention advice, social prescribing and signposting of available social services. Twenty-three (66%) patients were prescribed equipment to aid ADLs. The MDT recommended that advance care planning was considered for 20 (59%) patients. Despite 33 (94%) patients meeting the criteria for polypharmacy, a medication change was considered appropriate in only 2 (6%) patients. There was a higher proportion of dialysis-dependent patients experiencing problems with BADLs (71% vs 36%, *p* = 0.04) and nutrition (52% vs 14%, *p* = 0.02) than non-dialysis patients. There was also a higher proportion of dialysis-dependent patients who required the involvement of other healthcare professionals (62% vs 21%, *p* = 0.02) and further assessment or investigations (67% vs 21%, *p* = 0.01) than non-dialysis patients.

**Table 3:** Identified problems and recommended actions following Geriatric Assessment and multi-disciplinary team discussion

	Non-dialysis (*n* = 14)	Dialysis (*n* = 21)	All (*n* = 35)	*p* value
Identified problems, median (IQR)	3.5 (2.0)	6.0 (1.5)	5.0 (3.0)	0.007
Identified problems, *n* (%)				
Incontinence	2 (14)	5 (24)	7 (20)	0.68
Skin integrity	0 (0)	3 (14)	3 (9)	0.26
Falls risk	11 (79)	21 (100)	32 (91)	0.06
BADLs	5 (36)	15 (71)	20 (57)	0.04
IADLs	14 (100)	21 (100)	35 (100)	–
Depression/anxiety	1 (7)	8 (38)	9 (26)	0.06
Cognition	4 (29)	9 (43)	13 (37)	0.39
Nutrition	2 (14)	11 (52)	13 (37)	0.02
Social	1 (7)	1 (5)	2 (6)	1.00
Polypharmacy	13 (93)	20 (95)	33 (94)	1.00
Recommended actions, median (IQR)	3.0 (2.0)	5.0 (2.0)	4.0 (2.0)	0.004
Recommended actions, *n* (%)				
Health/social care advice	14 (100)	21 (100)	35 (100)	–
Equipment prescription	7 (50)	16 (76)	23 (66)	0.15
Medication change	0 (0)	2 (10)	2 (6)	0.51
Liaise with other health professionals	3 (21)	13 (62)	16 (46)	0.02
Further assessment/investigation	3 (21)	14 (67)	17 (49)	0.01
Falls clinic referral	4 (29)	12 (57)	16 (46)	0.10
Physiotherapy referral	3 (21)	5 (24)	8 (23)	1.00
Social care referral	1 (7)	4 (19)	5 (14)	0.63
Advance care planning	9 (64)	11 (55)	20 (59)	0.51

Data presented as number (%) or median (IQR)

*BADLs* basic activities of daily living, *IADLs* intermediate activities of daily living

## Discussion

This quality improvement project demonstrates the implementation of a novel care pathway for people living with frailty and CKD within a nephrology centre. Similar to previous reports [5], there was a high prevalence of frailty in those with CKD, with a third of all screened having a CFS score ≥ 5. This highlights the burden of frailty within our local CKD population, which prior to this project was most likely largely undetected. Each point increase in the CFS score was independently associated with an increased risk of hospitalisation and mortality. These findings are in keeping with previous studies that have evaluated the association between CFS scores and outcomes in those with CKD [25, 26]. Importantly, our results demonstrate that the CFS appropriately identified individuals at risk of adverse outcomes. It also highlights the pressing need for health services to offer interventions that aim to improve outcomes for this vulnerable patient group.

Geriatric impairments are associated with increased mortality risk in patients starting dialysis [27]. Within our cohort, there was a high prevalence of geriatric impairments identified during GA, suggesting appropriate patients were referred to the GA service. Notably, all patients experienced problems with IADLs. Previous studies have demonstrated a high prevalence of problems with IADLs in advanced CKD [28–30] and an association between problems with IADLs and hospitalisation and mortality [27]. Given the high prevalence of problems with IADLs, the high proportion of patients considered at risk of future falls is unsurprising. Falls are particularly concerning for those with advanced CKD, as they are associated with an increased risk of a fall-related injury compared with the non-CKD population [31] and associated with worse HRQOL [32]. Therefore, efforts to minimise future falls risk is of paramount importance for people living with frailty and CKD.

Patients receiving dialysis experienced more problems despite being younger than non-dialysis patients. Recommended actions reflected the problems identified, with more actions recommended for patients receiving dialysis. Studies have reported a high prevalence of geriatric impairments in patients receiving dialysis [28–30, 33, 34]. The increased prevalence of geriatric impairments in patients receiving dialysis emphasises the need for a holistic assessment, such as the CGA, in those with advanced CKD. Patients with CKD often prioritise outcomes relevant to maintaining independence and general well-being over prolonged survival [35, 36]. The identification of geriatric impairments prior to commencing dialysis treatment may influence shared-decision making between patients and nephrologists, particularly with respect to discussing the benefits and risks of dialysis treatment compared with conservative management. Further evaluation is needed to assess if interventions, such as the CGA, have a positive influence on this shared-decision making process.

There are acknowledged limitations to this quality improvement project, which is in an early stage of development. First, the cross-sectional analysis of the GA service does not allow conclusions to be made on overall benefit of the intervention. However, by implementing targeted interventions to mitigate the adverse outcomes associated with geriatric impairments, we hypothesise that there will be an associated improvement in the general health and well-being of patients. Second, validated measures were not used for all domains of the GA. The specialty group had concerns that patients would be at risk of respondent fatigue and therefore wanted to limit the number of questionnaires used to those that would be most clinically useful. A pragmatic decision was made for some domains to be assessed during the GA by an experienced OT, which reflects clinical practice. Third, a formal assessment of cognition was not used from the outset. The number of identified cases of cognitive impairment increased after introduction of the MoCA; in fact, all dialysis patients that performed a MoCA had a score ≤ 24. Therefore, the overall proportion of cognitive impairment reported is likely an under-estimation of the true prevalence of cognitive impairment in this cohort. However, this is a representative example of our PDSA approach. Finally, a validated tool was not used to assess problematic polypharmacy nor was polypharmacy assessed by a pharmacist, although medication appropriateness was assessed by a clinician.

This quality improvement project demonstrates the high burden of frailty and problems experienced by those with CKD. It also details an approach to the implementation of a frailty screening programme and GA service so that problems can be identified and a person-centred management plan developed. We encourage nephrology services to collaborate with local geriatric medicine departments and/or existing frailty teams to introduce quality improvement initiatives that aim to improve the care provided to patients living with frailty and CKD. Finally, we recommend further evaluation of the benefits of the CGA, particularly in relation to patient-reported outcomes, for patients with CKD and the impact that the CGA has on shared-decision making processes within advanced kidney care clinics.

## Electronic supplementary material

Below is the link to the electronic supplementary material.

Supplementary file1 (DOCX 289 kb)

## Data Availability

The datasets generated during and/or analysed during the current study are available from the corresponding author on reasonable request.
