# Supplementary material for: Implementation of a frailty screening programme and Geriatric Assessment Service in a nephrology centre: a quality improvement project
Source: J Nephrol. 2020 Oct 10;34(4):1215–24. doi: 10.1007/s40620-020-00878-y (PMC8357770; doi:10.1007/s40620-020-00878-y)
Supplement: Supplementary file 1 — Supplementary file1 (DOCX 289 kb) [file 40620_2020_878_MOESM1_ESM.docx]

**Supplementary Materials**

**Table 1. PDSA cycles**

| PDSA Cycle 1 |
| --- |
| Plan |
| - Proactively identify patients at risk of frailty and assess burden of frailty within the Department. |
| Do |
| - Introduce validated frailty screening tool, i.e. CFS. - Increase staff awareness of relevance of frailty for Renal Services and educate staff on how to use the CFS via formal presentations and ad-hoc one-on-one sessions. - Collaborate with a trust Data Analyst to support collection of hospitalisation and mortality data. |
| Study |
| - Process measure: number of patients screened each week. - Balancing measure: time to complete CFS assessments. - Outcome measure: distribution of CFS scores. - Outcome measure: hospitalisation/mortality risk stratified by CFS score (to establish if identifying at risk patients). |
| Act |
| - Incorporate the CFS within the established Holistic Care Tool used on outpatient haemodialysis units. - Incorporate documentation of CFS scores within the Electronic Patient Record System. - Develop an animated video to provide education on frailty screening and to describe the planned service. |
| PDSA Cycle 2 |
| Plan |
| - Offer holistic assessment for patients living with frailty and CKD. |
| Do |
| - Develop a multi-domain holistic assessment using principles of the CGA (termed GA) - Develop GA documentation. - Establish referral criteria for GA Service. |
| Study |
| - Process measure: number of patients referred for GA. - Balancing measure: time to complete GA. - Outcome measure: number of problems experienced by patients. - Outcome measure: type of geriatric impairments experienced by patients. |
| Act |
| - Referral criteria broadened. - Establish trust Renal Frailty MDT email account as single point of access for referrers. - Introduced telephone triage for patients referred for GA. - Incorporate MoCA routinely within GA. |
| PDSA Cycle 3 |
| Plan |
| Introduce regular MDT meetings to discuss GA performed to ensure person-centred care plan implemented. |
| Do |
| - Invite members of the wider Renal MDT to attend regular meetings. - Develop MDT meeting pro forma.   Coordinate regular MDT meetings. |
| Study |
| - Process measure: number of patients discussed during MDT meetings. - Outcome measure: number of recommended actions for patients.   Outcome measure: type of recommended actions for patients, including advance care planning. |
| Act |
| - Collaborate with trust End of Life Educator to provide staff training on advance care planning discussions. - Assess staff perceptions of the GA service.   Assess patient experience of the GA service. |

CFS, Clinical Frailty Scale. CKD, Chronic Kidney Disease. CGA, Comprehensive Geriatric Assessment. GA, Geriatric Assessment. MDT, Multi-Disciplinary Team. MoCA, Montreal Cognitive Assessment.


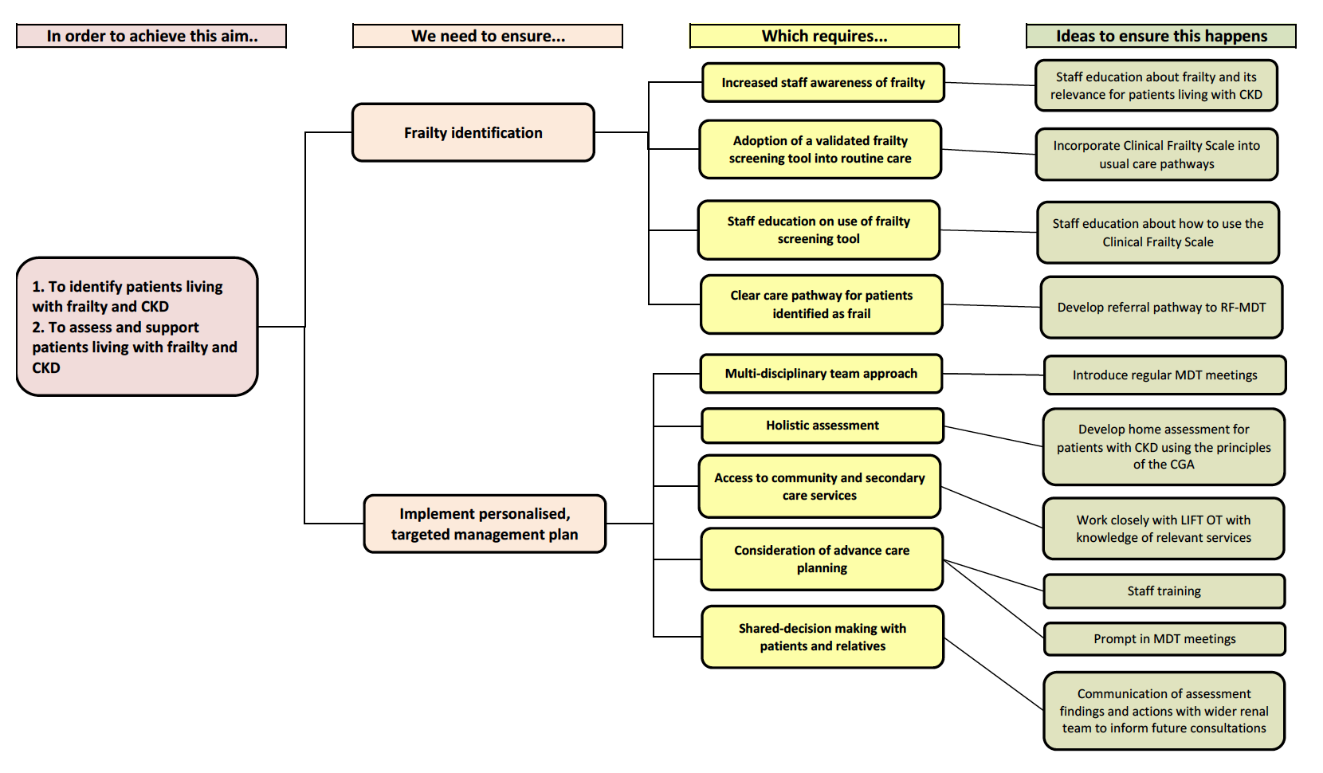


**Figure 1. Project driver diagram**
